# A Rare Case of Cutaneous Mucormycosis by *Syncephalastrum* Species in a Patient With Diabetes Mellitus: A Case Report and Review of the Literature

**DOI:** 10.1002/ccr3.72312

**Published:** 2026-03-16

**Authors:** Iqra Maryam, Khawaja Abdul Rehman, Aymen Shafqat, Obaid Ur Rehman, Omama Ayatullah, M Rafiqul Islam, Allahdad Khan

**Affiliations:** ^1^ Department of Medicine Fatima Jinnah Medical University Lahore Pakistan; ^2^ Department of Medicine CMH Lahore Medical College and Institute of Dentistry Lahore Pakistan; ^3^ Department of Medicine Services Institute of Medical Sciences Lahore Pakistan; ^4^ Department of Medicine Dow Medical College Karachi Pakistan; ^5^ Shaheed Suhrawardy Medical College Hospital Dhaka Bangladesh; ^6^ Department of Medicine Nishtar Medical University Multan Pakistan

**Keywords:** cutaneous mucormycosis, fungal infection, necrotizing soft tissue infection, syncephalastrum species

## Abstract

Infections caused by Syncephalastrum species in humans are relatively rare. However, clinicians must maintain a high suspicion for it in immunocompromised patients with cutaneous mucormycosis. Prompt histopathological and microbiological investigations, combined with timely antifungal therapy and surgical intervention, are crucial to prevent subsequent complications and amputations.

## 1 Introduction

Syncephalastrum species are environmental fungi commonly found in soil and decaying organic matter worldwide. Although infections are rare, they can occur in various settings, primarily affecting immunocompromised individuals with diabetes, underlying malignancy, chronic kidney and liver disease, and those who are on long‐term steroid therapy [1]. There have been isolated reports of cutaneous and nail infections, with a few cases resulting in pulmonary and rhinocerebral manifestations [2]. The rarity of these cases makes Syncephalastrum infections an underrecognized and poorly understood entity in clinical practice [3]. Syncephalastrum belongs to the order Mucorales. The pathogens most commonly responsible for human disease in the Mucorales order are Rhizopus, whereas other human pathogens include *Lichtheimia, Absidia, Mucor, Cunninghamella*, and *Apophysomyces spp* [4].

Diagnosing infections caused by Syncephalastrum can be difficult due to their nonspecific clinical presentation and the requirement for specialized fungal identification methods. A high index of suspicion in the right patient population is the key factor in diagnosis. Diagnostic procedures include biopsy and culture, which reveal aseptate hyphae and microsporangia [3]. Syncephalastrum species have aseptate hyphae that are broad and ribbon‐like, visible on histopathology stains, whereas wet mount microscopy of cultures might show a typical daisy head appearance [5]. In comparison with other mucorales species, Syncephalastrum species have smooth, pale appearing sporangiospores inside cylindrical finger‐like tubes called merosporangia that originate from the surface of a terminal vesicle [5]. The scarcity of documented cases in the literature also hinders the establishment of standardized treatment guidelines. Effective treatment options for Syncephalastrum infections primarily include the use of antifungal agents such as Amphotericin B or Posaconazole. Additionally, surgical debridement of nonviable tissue may be necessary to control the infection. It is also crucial to address and manage any underlying causes of immunosuppression to enhance the patient's ability to combat the infection [6].

This case report aims to broaden the existing understanding of Syncephalastrum infections by presenting a unique case of cutaneous foot involvement in a patient with uncontrolled diabetes. We will also discuss the challenges in diagnosis and management, highlighting the importance of early recognition and appropriate antifungal therapy.

## 2 Case History/Examination

**FIGURE 1:**
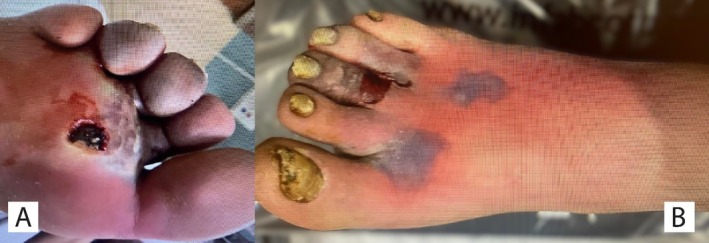
(A) Necrotic tissue on the plantar aspect of the foot; (B) Erythematous right foot with dorsal necrosis.

A 53‐year‐old Caucasian male, with a known history of diabetes, presented with a severe infection in his right foot characterized by redness, swelling, and discoloration for one week with no previous history of diabetic foot issues. The nails on his right toe showed signs of onychomycosis. A few centimeters wide necrotic tissue was present on the plantar surface (Figure 1A). Additionally, superficial skin necrosis was observed on the dorsum of the foot and at the base of the second and third toes (Figure 1B). Both dorsalis‐pedis and posterior tibial pulses were palpable, but the foot was cold to touch, suggesting compromised blood flow. Moreover, mild peripheral neuropathy was also present on examination. The remainder of the physical exam was unremarkable. Vitals were as follows: temperature 98.7°F, heart rate 106 beats per minute, respiratory rate 30 breaths per minute, and blood pressure 132/77 mmHg. There were no known drug or medication allergies. The patient denied any history of alcohol, tobacco, or illicit drug use. Family history was notable for diabetes mellitus, and immunization status was unknown at the time of evaluation. The patient's home medication included atorvastatin 80 mg once daily, metformin 500 mg once daily, insulin lispro 10 units subcutaneously with meals, insulin glargine 40 units subcutaneously at bedtime, ticagrelor 90 mg twice daily, and metoprolol succinate 12.5 mg twice daily for managing diabetes. He carried 4 g oral glucose tablets for the management of hypoglycemia. He also had a history of coronary artery disease, had undergone stent placement, and was on once daily acetylsalicylic acid 81 mg.

## 3 Differential Diagnosis, Investigations, and Treatment

**FIGURE 2:**
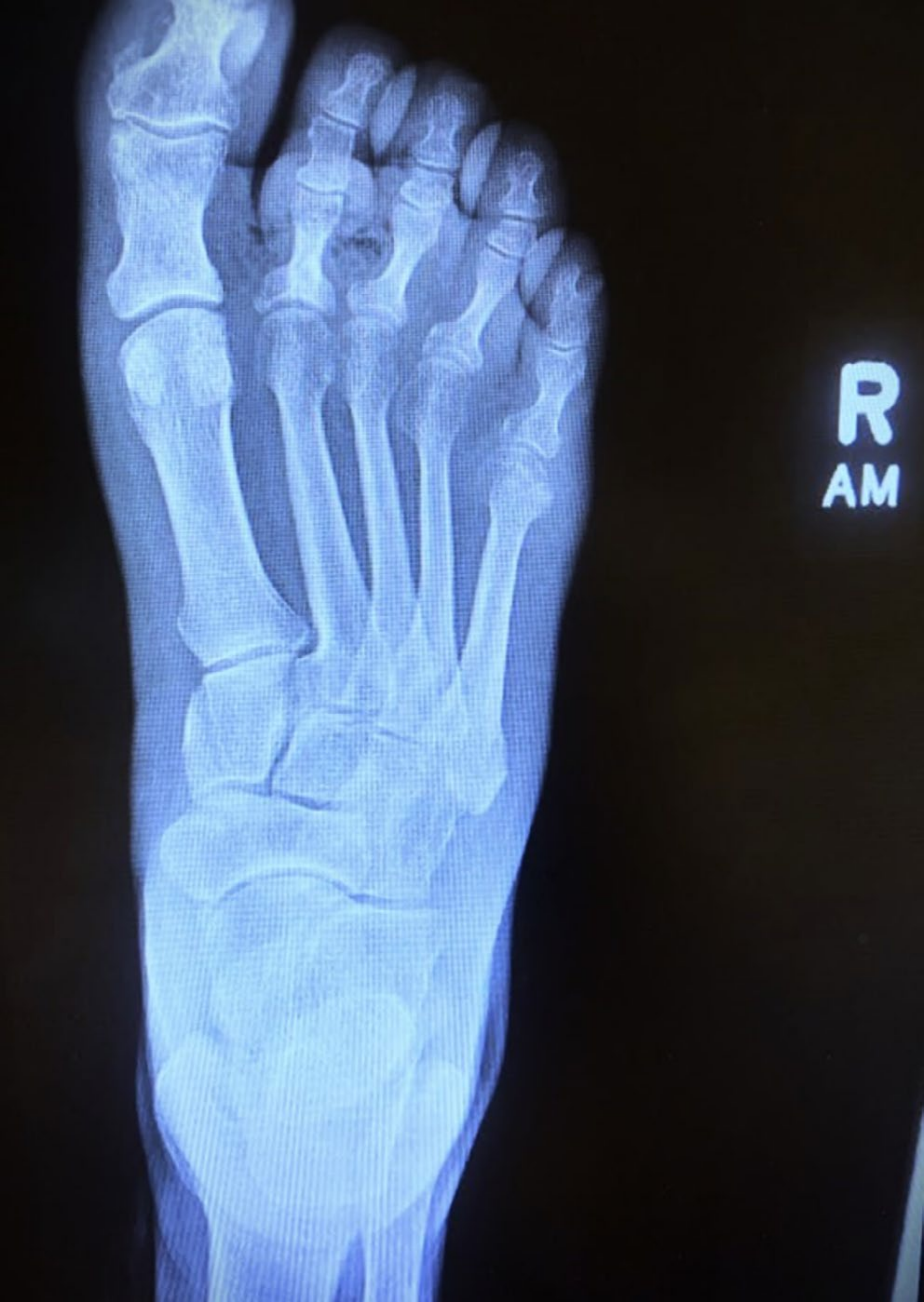
X‐Ray of right foot.

Laboratory tests revealed significantly elevated glucose levels (420 mg/dL) and an HbA1c of 14.7%, indicating poorly controlled diabetes mellitus. Hyponatremia (125 mmol/L) and hypochloremia (88 mmol/L) were also detected, possibly due to osmotic diuresis from hyperglycemia. Other laboratory investigations including serum potassium, CO_2_ (bicarbonates), blood urea nitrogen, creatinine and liver function tests were within normal limits. An X‐ray of the right foot was done which revealed gas locules in the soft tissue (Figure 2). Given that diabetic patients with foot ulcers are at an increased risk of developing osteomyelitis [7], a CT scan was ordered which showed no evidence of osteomyelitis.

**FIGURE 3:**
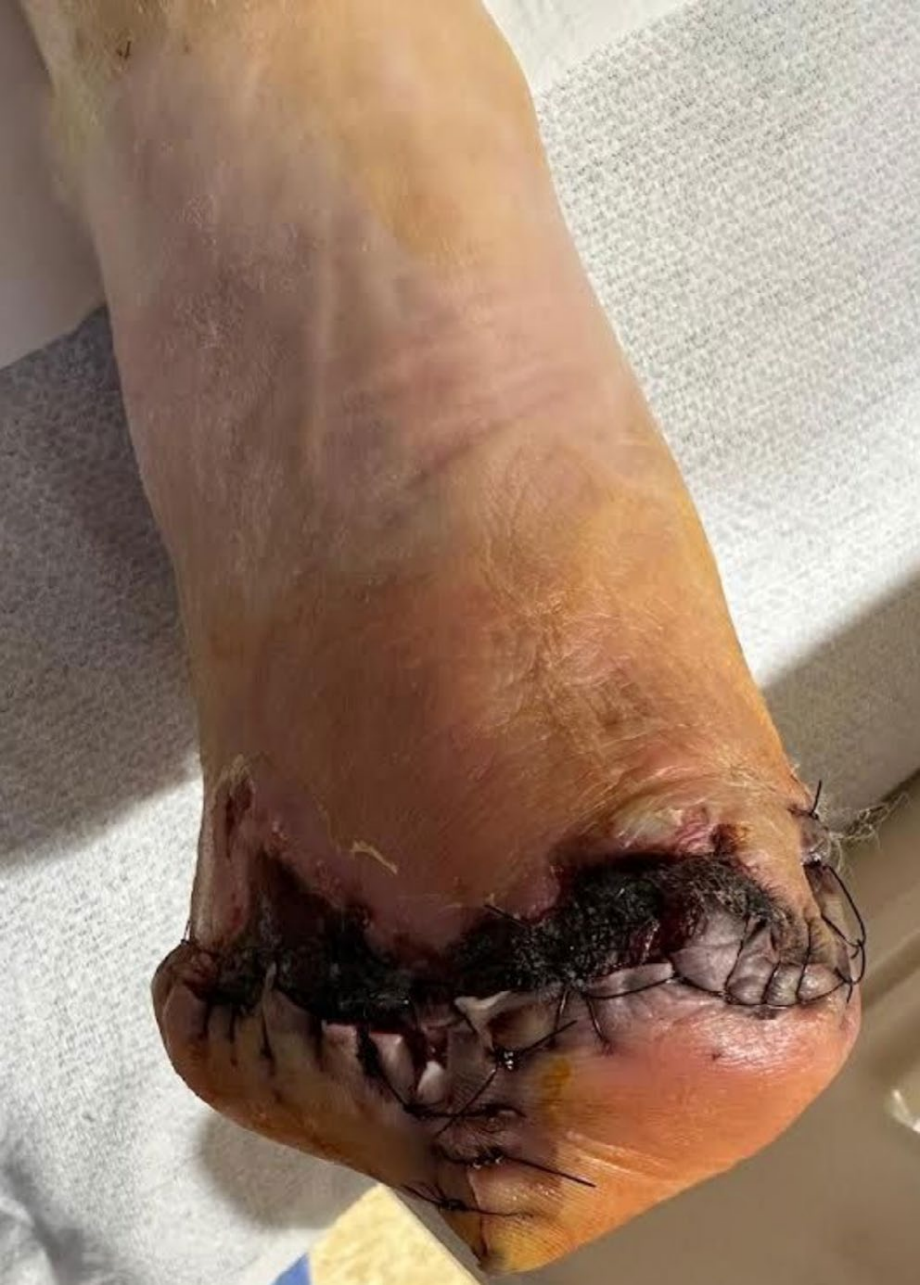
Post‐Transmetatarsal amputation condition.

**FIGURE 4:**
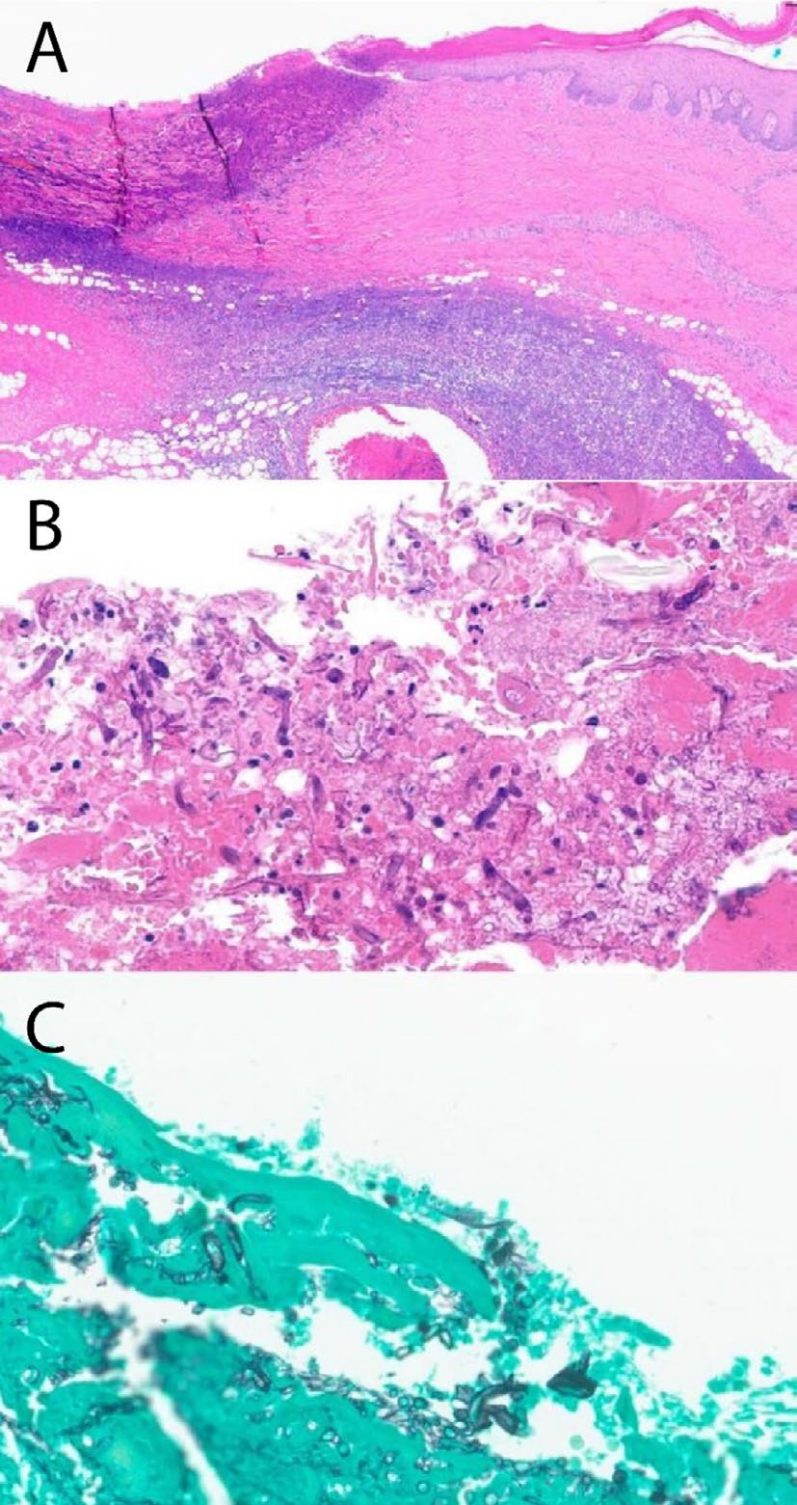
(A) 2 × magnification hematoxylin and eosin (H&E) staining demonstrates epidermal disruption with ulceration, along with dense inflammatory infiltrate in the dermis with extension into the subcutaneous tissue; (B) 40 × magnification H&E Staining shows fibrinoid necrosis, including neutrophils and other inflammatory cells, alongside the presence of fungal hyphae; (C) Grocott's methenamine silver stain demonstrates broad hyphae with ribbon‐like morphology, irregular branching, and lack of septations, consistent with mucormycetes.

The patient was initially suspected to have a polymicrobial diabetic foot infection and was empirically started on antibiotics, including vancomycin, clindamycin, cefepime, and metronidazole. He underwent amputation of the 2nd, 3rd, and 4th digits of the right foot, along with incision and debridement of multiple compartments, followed by a transmetatarsal amputation (TMA) two days later (Figure 3). A revision of the TMA was performed after a week. Culture results confirmed the presence of Enterococcus, Methicillin‐resistant *Staphylococcus aureus* (MRSA), and Syncephalastrum. Histopathology also confirmed the presence of Syncephalastrum species (Figure 4). Consequently, antibiotics were switched to linezolid, and liposomal Amphotericin B (AmB) was initiated as antifungal therapy. Regular debridement procedures were performed, accompanied by washouts with AmB to prevent the infection from spreading from the primary site. A detailed timeline of the interventions is provided in Table 1.

**TABLE 1:** Chronological timeline of interventions including antibiotics, antifungals, and surgical procedures.

Date	Interventions
10/06/2024	Admitted and started on vancomycin, clindamycin, cefepime, and metronidazole (initial impression polymicrobial bacterial infection)
11/06/2024	Amputation of the 2nd, 3rd, and 4th digits of the right foot, incision and debridement
13/06/2024	Transmetatarsal amputation (TMA) of right foot, initial cultures remained negative
16/06/2024	Right foot washout debridement and closure
19/06/2024	Culture results showed enterococcus, Methicillin‐resistant *Staphylococcus aureus* and Syncephalastrum, antibiotics changed to linezolid and started on Amphotericin liposomal
21/06/2024	Revision of TMA with Ampho wash out
25/06/2024	Further debridement
27/06/2024	Received 8 days of Ampho B liposomal and developed ARF
28/06/2024	Changed to Isovuconazole
01/07/2024	Culture remained positive for Syncephalastrum
03/07/2024	Started posaconazole due to presumed failure of Isovuconazole
08/07/2024	Lisfranc amputation and closure
10/07/2024	Wound clean. No necrosis and tolerated posaconazole, stopped linezolid

## 4 Outcome and Follow‐Up

**FIGURE 5:**
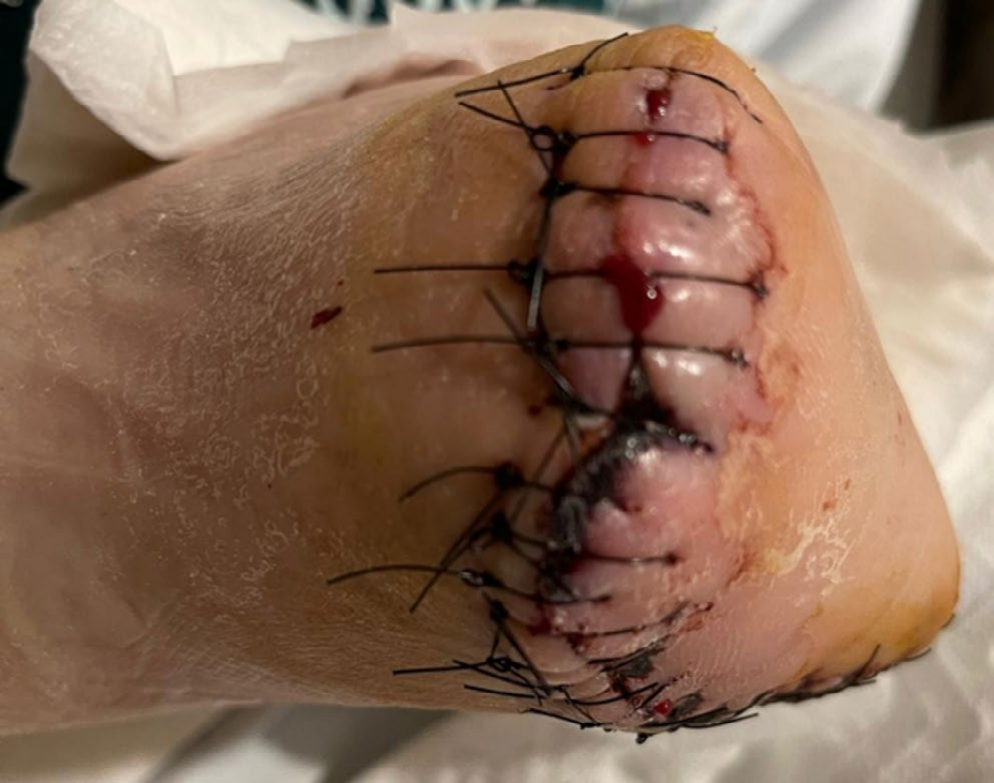
Patient's foot condition before discharge.

Following 8 days of AmB treatment, the patient developed acute renal failure (ARF), necessitating a switch to isavuconazole. Despite this, repeated cultures remained positive for Syncephalastrum, prompting a further change in antifungal therapy to posaconazole due to treatment failure with isavuconazole. The patient later underwent a Lisfranc amputation and showed no signs of necrosis afterward. Posaconazole was well tolerated, leading to the discontinuation of linezolid. Figure 5 shows the condition of the foot before he was discharged. The patient was followed for 2 months with no signs of worsening condition.

## 5 Discussion

Our study reports a case of necrotizing cutaneous mucormycosis of the right foot caused by Syncephalastrum sp. in an adult male with diabetes mellitus. Syncephalastrum, which belongs to the class Mucorales, is typically found in soil and decaying organic matter. While mucormycosis, also known as zygomycosis, is predominantly caused by other Mucorales species such as Rhizopus, Mucor, and Lichtheimia, Syncephalastrum is a rare human pathogen and accounts for only a few cases [8]. Mucormycosis can manifest in various forms, including rhinocerebral, pulmonary, cutaneous, gastrointestinal, disseminated, and miscellaneous infections [1]. Among these, cutaneous infections constitute 19%, making it the third most common type after rhinocerebral (39%) and pulmonary infections (24%) [2]. Syncephalastrum primarily affects immunocompromised individuals, such as those with malignancies, leukemia, severe diabetes mellitus, or neutropenia [1]. The fungus typically enters through inoculation, small skin injuries, or trauma. The majority of cases reporting Syncephalastrum infection were reported in India followed by the United States of America, Pakistan, and Egypt [9].

The cultures also detected MRSA and Enterococcus, both of which are known to cause subcutaneous and superficial skin infections as well as mucormycosis. These pathogens, in conjunction with Syncephalastrum, are associated with symptoms such as erythema, necrotic eschars, and ulcerations, which were observed in our patient [10]. Notably, MRSA often appears alongside Syncephalastrum, suggesting a connection in the disease's pathogenesis [11]. Additionally, elevated serum iron levels play a significant role in the pathogenesis of mucormycosis. Diabetic patients are particularly at risk due to ketoacidosis, which releases iron from binding proteins, resulting in high serum iron levels [12] and also impairs the phagocytic functions of neutrophils and macrophages [3]. Together, these factors create an environment conducive to fungal growth and disease development. One of our limitations is the inability to clearly delineate the relative contribution of the bacterial and fungal organisms identified in tissue samples. Although histopathology confirmed Syncephalastrum infection, management included both Amphotericin B and linezolid due to the presence of bacterial pathogens. Consequently, it is not possible to conclusively attribute the disease progression solely to the fungal pathogen.

A significant clinical finding in our patient was the onset of acute renal failure after 8 days of AmB treatment. This observation aligns with previously reported cases of Syncephalastrum infection treated with AmB, where a patient also developed acute kidney injury precisely after 8 days of treatment, as in our case [8]. This timing suggests a link to the nephrotoxic effects of AmB [13]. Despite AmB's widespread use due to its low minimum inhibitory concentration and minimal resistance development [14], its nephrotoxic properties must be managed. Recent research indicates that the addition of certain drugs and the use of novel nanoformulations may offer renal protection and mitigate the adverse effects of AmB [15, 16]. These newer approaches should be considered in place of traditional AmB for improved patient management.

There is limited literature on Syncephalastrum infections in humans. To date, fewer than 20 cases have been documented, including our patient and are listed in Table 2 (search string: (Mucormycosis OR Zygomycosis) AND (Syncephalastrum)) [5, 17, 18, 19, 20, 21, 22, 23, 24, 25, 26, 27, 28, 29]. Syncephalastrum is often found as a contaminant in various cultures, which can lead to missed diagnoses. In our case, repeated cultures consistently tested positive for Syncephalastrum, whereas a previously reported case showed that the fungus disappeared in follow‐up cultures after several weeks of antibiotic and antifungal treatment, raising questions about whether it was a contaminant or if the treatment was effective. Moreover, the initial cultures in our case were negative, complicating the diagnosis. This difficulty may stem from the low sensitivity and specimen‐handling issues associated with culture methods. As an aseptate fungi, Syncephalastrum is prone to cytoplasmic leakage, which can lead to death and prevent growth in culture [3]. The delay in antifungal treatment can significantly increase the risk of mortality, as a delay has been shown to double the risk [30]. In addition, histological examination revealed aseptate hyphae with microsporangia, confirming syncephalastrum's presence; however, this structure is also found in Aspergillus niger [31], which can complicate diagnosis. Further research is needed to develop more effective treatment and screening methods to improve patient management and reduce mortality.

**TABLE 2:** Summary of previously reported cases in the literature.

Year	Study	Site of infection	Comorbidity	Treatment/Management
1979	Kirkpatrick et al. [17]	Pulmonary	NA	NA
1980	Kamalam et al. [18]	Cutaneous, Osteomyelitis	Diabetes mellitus	Local debridement
2005	Schlebush et al. [5]	Intra‐abdominal	Immunocompetent	Surgical debridement
2006	Pavlović et al. [19]	Onychomycosis	Immunocompetent	Nail plate avulsion + topical nystatin
2008	Baradkar et al. [20]	Rhino‐orbito‐cerebral	Cirrhosis of liver	Surgical debridement
2010	Ramesh et al. [21]	Subcutaneous	Immunocompetent	Potassium iodide, itraconazole
2010	Amatya et al. [22]	Cutaneous	Immunocompetent	NA
2013	Mathuram et al. [23]	Rhino‐orbito‐cerebral	Diabetes mellitus	Amphotericin B
2014	Mangaraj et al. [24]	Subcutaneous tissue	Diabetes mellitus	Surgical debridement + Amphotericin B
2015	Baby et al. [25]	Onychomycosis	Diabetes mellitus	Surgical debridement + nystatin
2015	Rodriguez‐Gutierrez et al. [26]	Pulmonary	Non‐Hodgkin's lymphoma	Amphotericin‐B, itraconazole, caspofungin, voriconazole + surgical resection
2020	Irshad et al. [11]^a^	Pulmonary	Acute Myeloid Leukemia	Deoxycholate Amphotericin B + vancomycin
2020	Irshad et al. [11]^a^	Pulmonary	Chronic Obstructive Pulmonary Disease	Deoxycholate Amphotericin B + vancomycin
2020	Raju et al. [27]	Gastrointestinal	Diabetes mellitus	Surgical resection + Amphotericin B
2022	Armaki et al. [28]	Rhino‐orbital	Diabetes mellitus	Surgical debridement + Amphotericin B, posaconazole
2022	Mamali et al. [29]	Cutaneous	Open tibia fracture	Amphotericin B, voriconazole + surgical debridement
2026	Current study	Cutaneous	Diabetes mellitus	Surgical debridement + Amphotericin B, isavuconazole, posaconazole

^a^ Irshad et al. reported two cases.

Global guidelines recommend that most patients with zygomycosis require prompt medical or surgical intervention to control the disease's progression [6]. Previous studies indicate that surgical debridement followed by Amphotericin B (AmB) is the most common approach, along with addressing the underlying immunosuppressive conditions. Isavuconazole and posaconazole are suggested as salvage treatments [6]. In our case, the patient did not respond to isavuconazole, which may be attributed to the development of resistance to commonly used antifungals, as noted by Chowdhary et al. [32]. However, posaconazole was well‐tolerated and showed fewer adverse effects, demonstrating its effectiveness. This suggests that posaconazole can act as a potent extended‐spectrum agent when combined with surgical intervention in a case of Syncephalastrum‐related mucormycosis. Despite this, early diagnosis and treatment are essential for improving the disease's prognosis.

In conclusion, there is no definitive evidence guiding treatment strategies due to genetic variations and the absence of targeted testing. The management of such infection relies heavily on early diagnosis and timely treatment for the appropriate patient population. Clinicians should maintain a high level of suspicion for Syncephalastrum infection in patients presenting with cutaneous mucormycosis.

## Author Contributions

**Iqra Maryam:** conceptualization, data curation, writing – original draft. **Khawaja Abdul Rehman:** conceptualization, data curation, investigation, methodology, project administration, supervision, validation, visualization, writing – original draft, writing – review and editing. **Aymen Shafqat:** conceptualization, data curation, writing – original draft. **Obaid Ur Rehman:** validation, writing – original draft, writing – review and editing. **Omama Ayatullah:** writing – review and editing. **M Rafiqul Islam:** writing – review and editing. **Allahdad Khan:** writing – review and editing.

## Funding

The authors have nothing to report.

## Ethics Statement

The authors declare that written informed consent was obtained from the patient for publication of this manuscript and any accompanying images.

## Conflicts of Interest

The authors declare no conflicts of interest.

## Data Availability

The datasets used and/or analyzed during the current study are available from the corresponding author on reasonable request.
